# Equine Influenza Virus—A Neglected, Reemergent Disease Threat

**DOI:** 10.3201/eid2506.161846

**Published:** 2019-06

**Authors:** Alexandra Sack, Ann Cullinane, Ulziimaa Daramragchaa, Maitsetseg Chuluunbaatar, Battsetseg Gonchigoo, Gregory C. Gray

**Affiliations:** Institute of Veterinary Medicine, Ulaanbaatar, Mongolia (A. Sack, U. Daramragchaa, M. Chuluunbaatar, B. Gonchigoo);; Duke University, Durham, North Carolina, USA (A. Sack, G.C. Gray);; Irish Equine Centre, Johnstown, Ireland (A. Cullinane);; Duke-NUS Medical School, Singapore, Singapore (G.C. Gray);; Duke-Kunshan University, Kunshan, China (G.C. Gray)

**Keywords:** Equine influenza virus, influenza A, horses, zoonoses, H3N8, viruses

## Abstract

Equine influenza virus (EIV) is a common, highly contagious equid respiratory disease. Historically, EIV outbreaks have caused high levels of equine illness and economic damage. Outbreaks have occurred worldwide in the past decade. The risk for EIV infection is not limited to equids; dogs, cats, and humans are susceptible. In addition, equids are at risk from infection with avian influenza viruses, which can increase mortality rates. EIV is spread by direct and indirect contact, and recent epizootics also suggest wind-aided aerosol transmission. Increased international transport and commerce in horses, along with difficulties in controlling EIV with vaccination, could lead to emergent EIV strains and potential global spread. We review the history and epidemiology of EIV infections, describe neglected aspects of EIV surveillance, and discuss the potential for novel EIV strains to cause substantial disease burden and subsequent economic distress.

Equine influenza is a common, highly contagious respiratory disease of equids with a near-global distribution. Central Asia, Australia, and Japan experienced large equine influenza virus (EIV) outbreaks in 2007 (*1*,*2*). Serious outbreaks of EIV have occurred throughout history, causing substantial economic distress worldwide in the 19th and 20th centuries (*3*).

The most common clinical signs of EIV infection in equids are fever, lethargy, anorexia, nasal discharge, and a nonproductive dry cough (*4*). Mortality rates are generally low during EIV outbreaks; death is most common among foals or equids with preexisting poor health (*5*). Horses usually recover in 2 weeks with rest, but clinical signs, especially cough, can persist. EIV can result in a secondary bacterial bronchopneumonia, which can be fatal, particularly in young horses (*6*). Along with loss of life, the rest period required for equine recovery can cause economic hardship in areas where people rely on equids for income, such as for transportation or milk. EIV outbreaks also can disrupt economic drivers, such as the horse racing and show industries. EIV often is overlooked as a disease threat outside of equine communities, despite its known historical commercial importance and current zoonotic potential.

## Overview of Equine Influenza Viruses

EIVs are believed to have originated from avian influenza strains (*7*,*8*). Two subtypes, H7N7 and H3N8, historically have infected horses. EIV H7N7 was first recovered from horses in Europe during 1956 (*9*); it has not been isolated in horses since the 1970s, but serologic evidence suggests subclinical circulation through the 1990s (*7*,*8*). EIV H3N8 has 2 lineages, Eurasian and American. The American lineage includes Florida, Kentucky, and South America sublineages (*10*). The Florida sublineage is further divided into 2 antigenically distinct clades; both have been detected in Asia and Europe, but only clade 1 has been detected in North America. No Eurasian lineage viruses have been isolated since 2007, when it was detected in Switzerland (*11*). In 2016, all isolated EIV viruses were from the Florida lineage, clade 2 in Europe and clade 1 in the United States (*8*,*10*).

## Historical Impact

EIV-like equine respiratory diseases have been recorded since the 13th century (*9*). Although historical records predate human understanding of viral pathogens, they show EIV-like outbreaks preceded or, less commonly, followed several human influenza outbreaks. Despite the possibility that these illnesses were caused by pathogens other than EIVs (*3*), a review of records for 1688–1888 identified 56 years with documented outbreaks of influenza-like human or equine diseases in the Western Hemisphere (*3*). Records included 21 years in which both horses and humans were involved, 25 years with human-only involvement, and 10 years in which only horses were involved (*3*). Some of the largest assumed EIV outbreaks with smaller accompanying human influenza outbreaks occurred in 1727, 1750, 1760, and 1872 (*3*). EIV outbreaks generally occurred in spring or fall and were followed by similar human outbreaks 3 weeks later (*12*). Human influenza epidemics without equine infections were more common during winter months (*12*). Historical records provide a glimpse into a likely association between human and equine respiratory disease, but their reliability is limited by the lack of diagnostic testing. Others have provided more comprehensive reviews of these historical records (*3*,*12*,*13*).

A massive 1872 outbreak is considered the largest recorded EIV epizootic (*12*). Starting in Toronto in late September 1872, EIV spread along shipping routes across the United States and eventually into Central America and the Caribbean, stopping in Panama, which had no equine population to support EIV spread (*12*). Illness rates approached 100%, and the mortality rates were 2%–4% (*12*). Newspaper and veterinary reports from the time indicate travel, mail, and delivery of goods were severely hampered in the United States for weeks after the outbreak (*12*,*14*). This presumed outbreak of EIV also was blamed in part for a costly fire in Boston because fire wagons, pulled by young men instead of horses, could not reach the fire promptly (*12*,*14*). A mild human influenza was reported in people working with horses during the 1872 outbreak, but it is not known whether this was an EIV infection or human virus infection (*3*).

Major EIV outbreaks continued through the 1900s (Appendix Table). Many were associated with importation of horses, including outbreaks in South Africa in 1986, India in 1987, Hong Kong in 1992, and Dubai in 1995 (*15*). Earlier outbreaks in the 1990s might have gone unrecorded due to poor diagnostics or reporting mechanisms.

## Selected Outbreaks in the Past Decade

Influenza is endemic in horses in the United States and much of the world, with the exception of New Zealand and Iceland (*16*). Outbreaks of EIV infections in horses occurred globally throughout the 2000s. All inhabited continents had >1 country with an EIV outbreak during 2006–2017 (Figure) (*8*,*17*), and in 2015, China, France, Germany, Ireland, Malaysia, Sweden, the United Kingdom, and the United States experienced EIV outbreaks. Ireland, the United Kingdom, and the United States also had cases or outbreaks in 2016–2017 (*17*), as did China and Japan in 2017 (*8*,*17*). The United States recently experienced considerable EIV activity where the virus was detected in 23 states in 2015, 16 states in 2016, and 22 states in 2017 (*8*,*17*). 

**Figure:**
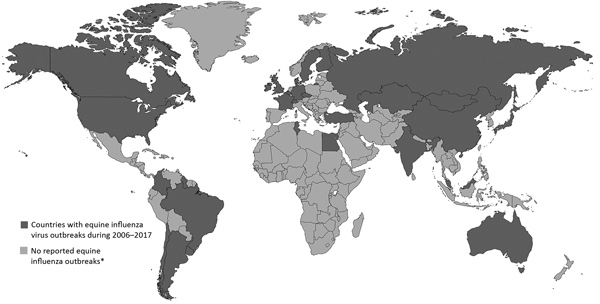
Equine influenza distribution map 2006–2015, compiled from Expert Surveillance Panel on Equine Influenza Vaccine Composition reports, 2006–2017 (*17*). Created with mapchart.net (http://www.mapchart.net). *No outbreaks occurred or no data reported.

Australia experienced a large outbreak in 2007, involving 70,000 horses living on >9,000 properties, that resulted in a 5% mortality rate (*18*,*19*). The virus was introduced by thoroughbred horses imported from Japan (*18*). Authorities believe EIV escaped a quarantine station due to lax biosecurity protocols. In addition, many horses certified as vaccinated against EIV had no protective antibodies, suggesting poor immune response, lack of vaccination compliance, or ineffective vaccines (*20*). Australia implemented an awareness and information campaign to supplement its interventions, which included quarantining, restricting horse movement, decontaminating properties, establishing disease control zones, and increasing surveillance and vaccination. The country was declared EIV-free in 2008 (*20*), but estimates of the economic cost to the equine industry are >$1 billion Aus (*18*).

During 2011–2012, an outbreak of EIV Florida clade 1 began in Chile and spread to multiple countries in South America (*4*) through the movement of horses in the rodeo and thoroughbred racing industries. EIV-positive horses were exported from Uruguay to Dubai (*4*), demonstrating the ease with which international transport could contribute to the spread of EIV. This importation was identified and stopped in the Dubai quarantine facility. Also during 2011–2012, a Florida clade 2 virus circulated in central Asia, Kazakhstan, western Mongolia, India, and western China (*1*).

In Mongolia, government records show 4 large EIV outbreaks with some of the highest known mortalities during 1974–1975, 1983–1984, 1993–1994, and 2007–2008 (*21*). Mortality rates of 20%–30% were reported for all but the 2007–2008 outbreak, during which the country implemented vaccination and the mortality rate decreased to ≈5% (*21*). Whereas some of these mortality rates are confounded partially by extreme weather and differences in reporting, diagnostics, and nutrition among horses, the reduced mortality rate seen with vaccination appears to be real. A 2012 EIV outbreak in Mongolia was linked to an outbreak in Kazakhstan that year and an outbreak in China in 2013 (*1*,*2*). Additional EIV outbreaks have occurred in Malaysia in 2015, the United States and Ireland in 2016, and Chile in 2018 (*8*,*17*). 

The primary focus of EIV prevention has been on domestic horses, but EIV is transmissible to all equids, including feral and wild herds, such as Przewalski’s horses. A 2007 outbreak in China affected ≈13,600 donkeys, resulting in 77 deaths. The donkeys had the typical clinical signs of EIV, and the most severe cases occurred in animals 6 months to 3 years of age (*22*). The Takhi herd of wild horses in China also experienced an illness rate near 100% and a 5% mortality rate (*23*) during that outbreak. Feral horses also were considered as a route of transmission during the 2007 outbreak in Australia, and the country set up a vaccination buffer zone. Although authorities considered the risk for infection low for feral horses, they considered risk to domestic horses from infected feral horses high (*24*).

## Potential for Cross-Species Spread

EIV infection is not limited to equids. Sporadic spillover of EIV to dogs has been detected in the United Kingdom and Australia (*25*–*27*). In the early 2000s, a canine H3N8 influenza of equine origin was discovered in the United States, where it continues to circulate with high transmission rates in dog shelters (*25*). Canine influenza in Australia occurred simultaneously with the 2007 EIV outbreak in horses (*26*). During 2004–2006, surveillance for influenza viruses in pigs in China discovered 2 H3N8 strains of equine origin (*28*). 

Cats experimentally infected with EIV demonstrated respiratory signs and virus shedding with transmission to other cats (*29*). A strain of influenza discovered in a camel in Mongolia was directly related to a circulating equine H3N8 strain, but horizontal spread in camels could not be determined (*30*). Although cross-species infections appear to be rare, horses potentially could play a role in the generation or amplification of a novel virus.

One of the most severe outbreaks of EIV occurred in China and involved an EIV related to an avian H3N8 strain. The outbreak had a mortality rate of ≈20% (*31*), but the virus was not sustained in the equine population (*32*). However, the close relationship between this EIV and avian H3N8 strains of the time (*31*,*32*) raises concerns that novel, highly virulent avian influenza viruses might adapt to horses and cause severe future mortality rates. 

In another instance in 2009, donkeys with moderate respiratory infection in a village in Egypt were found to have H5N1 during a simultaneous outbreak of highly pathogenic H5N1 in poultry (*33*). Historical reports in newspapers from the United States in 1872 describe a highly pathogenic disease in poultry that seems clinically similar to modern avian influenza viruses (*12*). Although the connection of this avian disease to the 1872 EIV epizootic is unknown, local newspaper reports linked it with the movement of infected horses (*12*), suggesting equids could be a potential reservoir or amplifying host for future avian influenza infections.

Humans are also a potential host for EIV. Experimental infection of antibody-negative human volunteers in the 1960s saw >60% of them seroconvert and have positive virus cultures from throat swabs collected 2–6 days after nasal inoculation. Most of the human volunteers also shed virus from day 2 through day 5 but rarely shed past day 6 (*34*,*35*). In the same study, horses became infected by strains of EIV passed through humans (*34*). During 1958–1963, human serum samples were tested in the Netherlands for EIV antibodies. Less than 0.5% of people <60 years of age had elevated antibody titers, but 11.5% of people >60 years of age had elevated EIV antibodies, with >40% EIV antibody elevation among people >70 years of age. The authors surmised that a virus resembling the 1963 EIV strain infected humans during 1896–1900 (*36*). The study was performed before the human H3 influenza virus was recorded and determined to have crossed from ducks to humans in 1965, although the equine H3 strain is older (*37*). The evidence suggests past equine-to-human interspecies transmission.

Studies of humans exposed to horses during the 2007 EIV outbreak in Australia found only 10% of people had serologic reaction against EIV, all at a low level, suggesting cross-reactivity with human influenza strains (*38*). A similar study in Mongolia found only 4.8% of people tested had elevated EIV antibodies, all at low titer levels that could be explained by cross-reactivity with seasonal human influenza virus infection or vaccine (*39*). However, a study in Iowa using new serologic assays found that people regularly exposed to horses were more likely to have elevated antibodies against EIV than people not exposed to horses in the previous 10 years (*40*). Smoking was an added risk factor for elevated antibodies against EIV, possibly because of oral contamination from touching horses before smoking or because of a compromised immune system from smoking (*40*). A single probable horse-to-human EIV transmission case was observed in Chile during 1973, but the human’s influenza virus was not typed (*41*). Although experimental infection of humans has been demonstrated, evidence for natural infection is more equivocal.

## Environmental Transmission

EIV has multiple potential direct and indirect routes of transmission and is especially communicable when it enters a large, previously unexposed population of equids. Research is clarifying the role of the environment and weather on EIV transmission. During the 2007 outbreak in Australia, wind speeds >30 kph from the direction of infected horses correlated with an increased risk for infection for horses downwind (*19*). Humidity and temperature also might have contributed to EIV transmission (*19*). These findings match results from a laboratory study that found enhanced aerosolizing of influenza virus with colder and drier conditions (*42*). Further analysis in Queensland, Australia, found east to west spread of EIV with distances of 1–2 km consistent with wind patterns (*43*). In addition, some avian influenza virus strains persist in water and remain infectious for >2 months (*44*), implying water also is a potential source of transmission for EIV. 

## Vaccination and Prevention Strategies

The World Organisation for Animal Health (OIE) recommends current EIV vaccines include both Florida clade 1 and clade 2 H3N8 strains (*8*). Because EIV H7N7 has not been isolated since the late 1970s and the Eurasian strain of H3N8 has not been seen since 2007 (*8*,*11*), OIE suggests omitting these from equine influenza vaccines. The American Association of Equine Practitioners recommends EIV vaccination unless a horse is in a closed and isolated facility and that high-risk populations, including young show and race horses, be vaccinated every 6 months (*16*). Horse shows and races have been recognized mechanisms of increased dispersal and spread of EIV since a 1963 EIV outbreak across the continental United States linked to infected horses imported by air from Argentina into Florida (*45*). Most EIV infections in the United States in 2017 were in show and race horses with an unknown vaccination history (*2*,*8*,*20*). As the number of horses transported by air for breeding or racing increases, so does the probability of future EIV outbreaks among horses (*15*).

Because previously vaccinated horses shed virus without clinical signs, EIV vaccine is useful for reducing clinical signs and reducing virus shed but does not eliminate potential transmission (*20*,*46*). In 2003, an outbreak of EIV closely related to the Kentucky sublineage occurred in regularly vaccinated thoroughbred horses in England (*47*). Before this outbreak, the American strain included in the vaccine was considered sufficient to protect horses. This outbreak helped redefine how antigenic drift reduces the efficacy of EIV vaccinations (*47*). A study of vaccine containing H3N8 A/eq/Kentucky/98 found >40% of vaccinated ponies shed EIV after experimental challenge with aerosolized EIV, although for fewer days than animals in an unvaccinated control group (*46*). 

More effective vaccines are needed, especially considering the role failed vaccination potentially played in the 2007 outbreak in Australia (*20*). Even while the OIE recommends all vaccines contain Florida clade 1 and clade 2 virus, many vaccines contain outdated strains (*8*). A review of current vaccinations found that many commercial vaccines are not updated until a major outbreak, such as the one in Australia (*48*).

Because vaccination does not prevent viral shedding, good management practices are imperative for preventing EIV outbreaks (*46*). Before reaching its goal of 80%–90% EIV vaccination rates in high-risk areas, Australia saw a decline in new cases likely related to adoption of movement restrictions and biosecurity protocols (*49*). Ring vaccination, however, is credited for stopping the spread and reducing risk for EIV infection so normal equine activities could be resumed (*49*). 

Preventive measures should include international surveillance and investigation of vaccine failure (*8*). Veterinarians can further help educate owners in order to help improve surveillance, diagnostics, and increase EIV vaccination (*15*).

## Surveillance Efforts

After major EIV outbreaks in 1989 related to outdated vaccine strains, the OIE Biological Standards Commission initiated the formal global Equine Influenza Surveillance Programme in 1995 (*50*). OIE currently has reference laboratories in Ireland, the United Kingdom, and the United States. Additional laboratories in Asia, Europe, and South America collect data on outbreaks of EIV and strain characterization, which the Expert Surveillance Panel on Equine Influenza Vaccine Composition reviews annually. The panel, composed of OIE and World Health Organization representatives, makes recommendations on vaccine updates, which it publishes in an annual bulletin (*8*).

The level of active or passive EIV surveillance in each country depends on the nature of the horse industry, status of the disease, laboratory capability, and financial resources available for veterinary intervention. Many laboratories involved in EIV surveillance experience difficulty obtaining sufficient samples because horse owners seldom request a confirmatory diagnosis for influenza (*15*). Surveillance is compounded by the failure of some diagnostic laboratories to characterize virus or to submit positive real-time PCR samples to an OIE reference laboratory for virus isolation and antigenic characterization.

OIE continues to develop global EIV surveillance through its Laboratory Twinning Programme (http://www.oie.int/support-to-oie-members/laboratory-twinning), which transfers skills and reagents from OIE reference laboratories to other institutes to increase their capacity to implement effective surveillance programs and contribute data. Recently, the OIE reference laboratory at the Animal Health Trust (Suffolk, UK) completed a 3-year twinning project with the National Research Centre on Equines in Hisar, India. In 2016, the OIE reference laboratory at the Irish Equine Centre (Johnstown, Ireland) completed a twinning project with Harbin Veterinary Institute in Harbin, China. Currently, no OIE reference laboratory for equine influenza exists outside of Europe or North America, but OIE anticipates that twinning will help a laboratory in Asia meet the necessary requirements, develop a regional network, and provide diagnostic support to neighboring countries. At the time of our report, the surveillance program reports that Australia and New Zealand are EIV free and that a large EIV outbreak with fatalities in donkeys has been reported in Africa, but there is a lack of sufficient information from many countries in the Middle East and elsewhere EIV may be circulating (http://www.oie.int/wahis_2/public/wahid.php/Reviewreport/Review/viewsummary?reportid=29135).

## Future Challenges

Equine influenza is a highly contagious virus with the potential to cause global harm. The 2007 EIV outbreak in Australia demonstrated the economic impact the virus can have when introduced into a previously unexposed equine population (*18*). Furthermore, potential novel and virulent avian influenza virus strains could cross into horses and rapidly spread despite previous equid vaccinations (*31*,*33*). Risk from avian strains is compounded by EIV’s potential for infecting humans (*13*). Although the role of humans in EIV evolution is unknown, historical and serologic evidence suggests EIV has zoonotic potential and is known to infect other nonhuman species (*26*,*28*,*30*). Historical review suggests the 1889 human influenza pandemic might have been of equine origin, with equids playing the role that swine play in modern outbreaks (*12*). With all this in mind, we posit that EIV should be recognized as a potential epidemic, if not pandemic, threat.

Appendix Timeline of major equine influenza outbreaks in the 20th and 21st centuries.
