# Supplementary material for: Equine Influenza Virus—A Neglected, Reemergent Disease Threat
Source: Emerg Infect Dis. 2019 Jun;25(6):1185–91. doi: 10.3201/eid2506.161846 (PMC6537720; doi:10.3201/eid2506.161846)
Supplement: Appendix — Timeline of major equine influenza outbreaks in the 20th and 21st centuries. [file 16-1846-Techapp-s1.pdf]

DOI: https://doi.org/10.3201/eid2506.161846

Equine Influenza Virus–Historical Perspective on a Neglected, Reemergent Disease Threat

Appendix.

Appendix Table. Timeline of major equine influenza outbreaks in the 20th and 21st Centuries

Country	Year
United States and Canada (*1*)	1963
Japan (*2*)	1971
Malaysia (*3*)	1977
United Kingdom (*4*)	1979
South Africa (*5*)	1986
India (*6*)	1987
United Kingdom (*4*)	1989
China (*7*)	1989
Nigeria (*8*)	1991
Hong Kong (*9*)	1992
United States (*10*)	2004
Australia (*11*)	2007
India and central Asia (*12*)	2008
South America and central Asia (*12*,*13*)	2011–2012

References

1. Scholtens RG, Steele JH, Dowdle WR, Yarbrough WB, Robinson RQ. U.S. Epizootic of equine influenza, 1963. Public Health Rep. 1964;79:393–402. PubMed http://dx.doi.org/10.2307/4592142

2. Goto H, Shimizu K, Abe T, Kanamitsu M. Sero-epidemiological study on equine influenza in Japan. J Clin Microbiol. 1976;2:89–93. PubMed

3. Sreenivasan CC, Jandhyala SS, Luo S, Hause BM, Thomas M, Knudsen DEB, et al. Phylogenetic analysis and characterization of a sporadic isolate of equine influenza A H3N8 from an unvaccinated horse in 2015. Viruses. 2018;10:31. PubMed http://dx.doi.org/10.3390/v10010031

4. Livesay GJ, O'Neill T, Hannant D, Yadav MP, Mumford JA. The outbreak of equine influenza (H3N8) in the United Kingdom in 1989: diagnostic use of an antigen capture ELISA. Vet Rec. 1993;133:515–9. PubMed http://dx.doi.org/10.1136/vr.133.21.515

5. Kawaoka Y, Webster RG. Origin of the hemagglutinin on A/Equine/Johannesburg/86 (H3N8): the first known equine influenza outbreak in South Africa. Arch Virol. 1989;106:159–64. PubMed http://dx.doi.org/10.1007/BF01311048

6. Uppal PK, Yadav MP, Oberoi MS. Isolation of A/Equi-2 virus during 1987 equine influenza epidemic in India. Equine Vet J. 1989;21:364–6. PubMed http://dx.doi.org/10.1111/j.2042-3306.1989.tb02690.x

7. Guo Y, Wang M, Kawaoka Y, Gorman O, Ito T, Saito T, et al. Characterization of a new avian-like influenza A virus from horses in China. Virology. 1992;188:245–55. PubMed http://dx.doi.org/10.1016/0042-6822(92)90754-D

8. Adeyefa C, James M, McCauley J. Antigenic and genetic analysis of equine influenza viruses from tropical Africa in 1991. Epidemiol Infect. 1996;117:367–374. PubMed

9. Powell DG, Watkins KL, Li PH, Shortridge KF. Outbreak of equine influenza among horses in Hong Kong during 1992. Vet Rec. 1995;136:531–6. PubMed http://dx.doi.org/10.1136/vr.136.21.531

10. Crawford PC, Dubovi EJ, Castleman WL, Stephenson I, Gibbs EPJ, Chen L, et al. Transmission of equine influenza virus to dogs. Science. 2005;310:482–5. PubMed http://dx.doi.org/10.1126/science.1117950

11. Watson J, Daniels P, Kirkland P, Carroll A, Jeggo M. The 2007 outbreak of equine influenza in Australia: lessons learned for international trade in horses. Rev Sci Tech. 2011;30:87–93. PubMed http://dx.doi.org/10.20506/rst.30.1.2021

12. Karamendin K, Kydyrmanov A, Kasymbekov Y, Khan E, Daulbayeva K, Asanova S, et al. Continuing evolution of equine influenza virus in central Asia, 2007–2012. Arch Virol. 2014;159:2321–7. PubMed http://dx.doi.org/10.1007/s00705-014-2078-3

13. Perglione CO, Gildea S, Rimondi A, Miño S, Vissani A, Carossino M, et al. Epidemiological and virological findings during multiple outbreaks of equine influenza in South America in 2012. Influenza Other Respi Viruses. 2016;10:37–46. PubMed http://dx.doi.org/10.1111/irv.12349
